# Effect of Polyethylene Glycol on the Formation of Magnetic Nanoparticles Synthesized by *Magnetospirillum magnetotacticum* MS-1

**DOI:** 10.1371/journal.pone.0127481

**Published:** 2015-05-20

**Authors:** Hirokazu Shimoshige, Hideki Kobayashi, Toru Mizuki, Yutaka Nagaoka, Akira Inoue, Toru Maekawa

**Affiliations:** 1 Bio-Nano Electronics Research Centre, Toyo University, Kawagoe, Saitama, Japan; 2 Japan Agency for Marine-Earth Science and Technology, Yokosuka, Kanagawa, Japan; 3 Graduate School of Interdisciplinary New Science, Toyo University, Kawagoe, Saitama, Japan; Louisiana State University and A & M College, UNITED STATES

## Abstract

Magnetotactic bacteria (MTB) synthesize intracellular magnetic nanocrystals called magnetosomes, which are composed of either magnetite (Fe_3_O_4_) or greigite (Fe_3_S_4_) and covered with lipid membranes. The production of magnetosomes is achieved by the biomineralization process with strict control over the formation of magnetosome membrane vesicles, uptake and transport of iron ions, and synthesis of mature crystals. These magnetosomes have high potential for both biotechnological and nanotechnological applications, but it is still extremely difficult to grow MTB and produce a large amount of magnetosomes under the conventional cultural conditions. Here, we investigate as a first attempt the effect of polyethylene glycol (PEG) added to the culture medium on the increase in the yield of magnetosomes formed in *Magnetospirillum magnetotacticum* MS-1. We find that the yield of the formation of magnetosomes can be increased up to approximately 130 % by adding PEG200 to the culture medium. We also measure the magnetization of the magnetosomes and find that the magnetosomes possess soft ferromagnetic characteristics and the saturation mass magnetization is increased by 7 %.

## Introduction

Magnetotactic bacteria (MTB) are Gram-negative prokaryotes that synthesize intracellular magnetic nanoparticles named magnetosomes. Magnetosomes are membrane-bounded crystals, which are composed of either magnetite (Fe_3_O_4_) or greigite (Fe_3_S_4_) and characterized by the narrow size distribution in each cell ranging from 30 to 120 nm, distinct species-specific crystal morphology and chemical purity, form aligned structures, arranging a single or multiple linear chains within the cells [1–6]. Magnetosomes are formed via some biomineralization process with strict control over the chemical composition, morphology, size, and intracellular localization of magnetic minerals. The magnetosome crystals, which are synthesized in the magnetosome membrane vesicles, are covered with lipid bilayer membranes containing various types of proteins. Thanks to the unique characteristics of magnetosomes, MTB are of great interest and importance, considering a number of potential applications of them to the biomedical and environmental studies such as drug carriers [7,8], immunoassays [9,10], cell separation [11], enzyme immobilization [12], gene delivery systems [13], and mineral recovery systems [14]. However, the above technologies have not yet been fully developed even at an academic level, let alone on a commercial scale since it is still extremely difficult to grow and produce high yields of magnetosomes under the present growth conditions [15]. Most of the studies on the formation of magnetosomes have focused on three strains of genus *Magnetospirillum* such as *M*. *magnetotacticum* MS-1, *M*. *magneticum* AMB-1, and *M*. *gryphiswaldense* MSR-1 [15–17]. It is supposed that the alteration of substances in the culture medium may change the biomineralization process. There have been quite a few studies aiming at the enhancement of the growth rate of *Magnetospirillum* and formation of magnetosomes, altering the environmental conditions such as the pH and the concentration of oxygen, and adding salt, and some amino acids and proteins to the culture medium [16–21]. Here, we investigate as a first attempt the effect of polyethylene glycol (PEG) added to the culture medium on the increase in the yield of magnetosomes formed in *M*. *magnetotacticum* MS-1. We find that the yield of the formation of magnetosomes can be increased up to approximately 130% by adding PEG200 to the culture medium. The magnetosomes show soft ferromagnetic characteristics and the saturation mass magnetization of MS-1 is increased by 7%.

## Materials and Methods

We obtained *Magnetospirillum magnetotacticum* MS-1 (JCM21281^T^) from the Japan Collection of Microorganisms. MS-1 was grown in 45 ml of the conventional Magnetospirillum medium (DSMZ 380). Importantly, 10 mM l^-1^ of HEPES was added to the conventional culture medium so that the pH of the culture was kept constant at 7.0. 25 μM of ferric quinate, which had been prepared by mixing 4.5 mg ml^-1^ of FeCl_2_•6H_2_O and 1.9 mg ml^-1^ of quinic acid with 1.0 l of distilled water, was added to the culture medium. MS-1 cells were cultured in liquid medium under an O_2_ (1%)-N_2_ (99%) atmosphere at 28°C in the dark. The microaerobic conditions were achieved by sparging the culture medium with a mixture of O_2_ (1%)-N_2_ (99%) gases through a butyl rubber stopper at a volume flow rate of 0.1 l min^-1^ for 10 min. The inoculum used for the initiation of cultures was grown by three sequential transfers at a ratio of 10% (vol/vol).

First of all, the effect of organic solvent, surfactant, fatty acid, and polysaccharide substances on the growth of MS-1 cells and the formation of magnetosomes was investigated as a preliminary experiment by adding individually 1 ml of *n*-dodecane, 1 ml of *n*-nonane, 1 ml of *n*-octane, 1 ml of cyclooctane, 1 ml of diphenylether, 1 ml of *n*-hexane, 0.01% (vol/vol) *n*-octanol, 0.5 and 1.0% (vol/vol) PEG6,000, 0.5 and 1.0% (vol/vol) Triton-X100, 0.5 and 1.0% (vol/vol) oleic acid, 0.5 and 1.0% (vol/vol) olive oil, 0.5 and 1.0% (vol/vol) glycerol, 0.1% (wt/vol) soluble starch, 0.1% (wt/vol) carboxymethylcellulose (CMC), and 0.1% (wt/vol) pectin to the modified Magnetospirillum medium (see S1 Text and S1 Table). Based on the result obtained by the above preliminary experiment, the effect of the molecular weight of PEGs, in particular, on the growth of MS-1 cells and the formation of magnetosomes was investigated, adding 0.5% (vol/vol) PEG200, 0.5% (vol/vol) PEG6,000, 0.5% (vol/vol) PEG20,000, and 0.5% (wt/vol) PEG500,000 to the culture medium.

We evaluated the bacterial cell density in the culture medium, measuring the absorbance of photons of 600 nm wavelength using a spectrometer (DU730, Beckman Coulter). The number of bacterial cells (cells ml^-1^) was also counted using a Bacteria Counting Chamber (Erma).

The response of MTB grown in the culture medium to an external magnetic field was checked by an optical microscope (DM5000B, LEICA) using a ferrite magnet (150 mm × 100 mm × 25.4 mm) (Niroku Seisakusho).

MS-1 cells were harvested at the stationary phase by centrifugation at 5,000 × *g* for 30 min. The pellets obtained after centrifugation were washed with 1 ml of 10 mM HEPES buffer (pH7.0) and centrifuged again at 5,000 × *g* for 10 min. The supernatant was removed and the pellets were resuspended in 2.5% glutaraldehyde with 10 mM HEPES buffer (pH7.0) overnight at 28°C to fix the cells. After fixation, the cells were washed with 1 ml of 10 mM HEPES buffer (pH7.0) and centrifuged at 5,000 × *g* for 10 min and the supernatant was removed. The above washing procedure was repeated three times. The washed cells were stored at 4°C. The cells were placed on TEM grids (200-mesh Cu Formvar/carbon-coated grid, JEOL) and rinsed three times with sterile distilled water. We observed the bacterial cells with a TEM (JEM-2100, JEOL) and the number of magnetosomes in each cell was counted targeting 130 individual cells, which had been grown under the same growth conditions. The size of magnetosomes was also measured targeting at least 1,000 magnetosomes, which had been formed under the same experimental conditions.

Bacterial cells were washed three times with sterile distilled water and collected by centrifugation at 5,000 × g for 10 min at 4°C and stored at -80°C. The pellets were freeze-dried in a vacuum chamber for the measurement of magnetization. Powder capsules (P125E, Quantum Design), into which the samples had been introduced, were mounted in brass sample holders and the magnetization of magnetosomes was measured by a superconducting quantum interference device (SQUID) magnetometer (MPMS3, Quantum Design) at 300 K. The external magnetic field was changed at the following speeds during the measurement; 10 Oe s^-1^ from 0 to 100 Oe, 100 Oe s^-1^ from 100 to 1000 Oe, and 500 Oe s^-1^ from 1000 to 5000 Oe, where 1 Oe = 10^3^/(4π) A m^-1^.

## Results and Discussion

In order to investigate the effect of organic solvent, surfactant, fatty acid, and polysaccharide on the growth of MS-1 cells and the formation of magnetosomes, strain MS-1 was grown in the presence of various additional substances as preliminary experiments as described above (see S1 Table), assuming that they may affect the structures of the membranes of the cells and/or magnetosomes and as a result, the growth of MS-1 cells and the formation of magnetosomes may be altered. The result of the preliminary experiments is summarized in S1 Text and S1 Table. Among the substances added to the culture medium, 0.5% PEG6,000 was most effective in the growth of MS-1 and the formation of magnetosomes (see S1 Table, S2 Text and S1 Fig), which suggested that PEG molecules may act positively in the synthetic process of magnetosomes. We therefore added PEG molecules of different molecular weights to the culture medium, fixing the concentration of each PEG at 0.5%, and observed the growth of MS-1 and the formation of magnetosomes.

**Fig 1:**
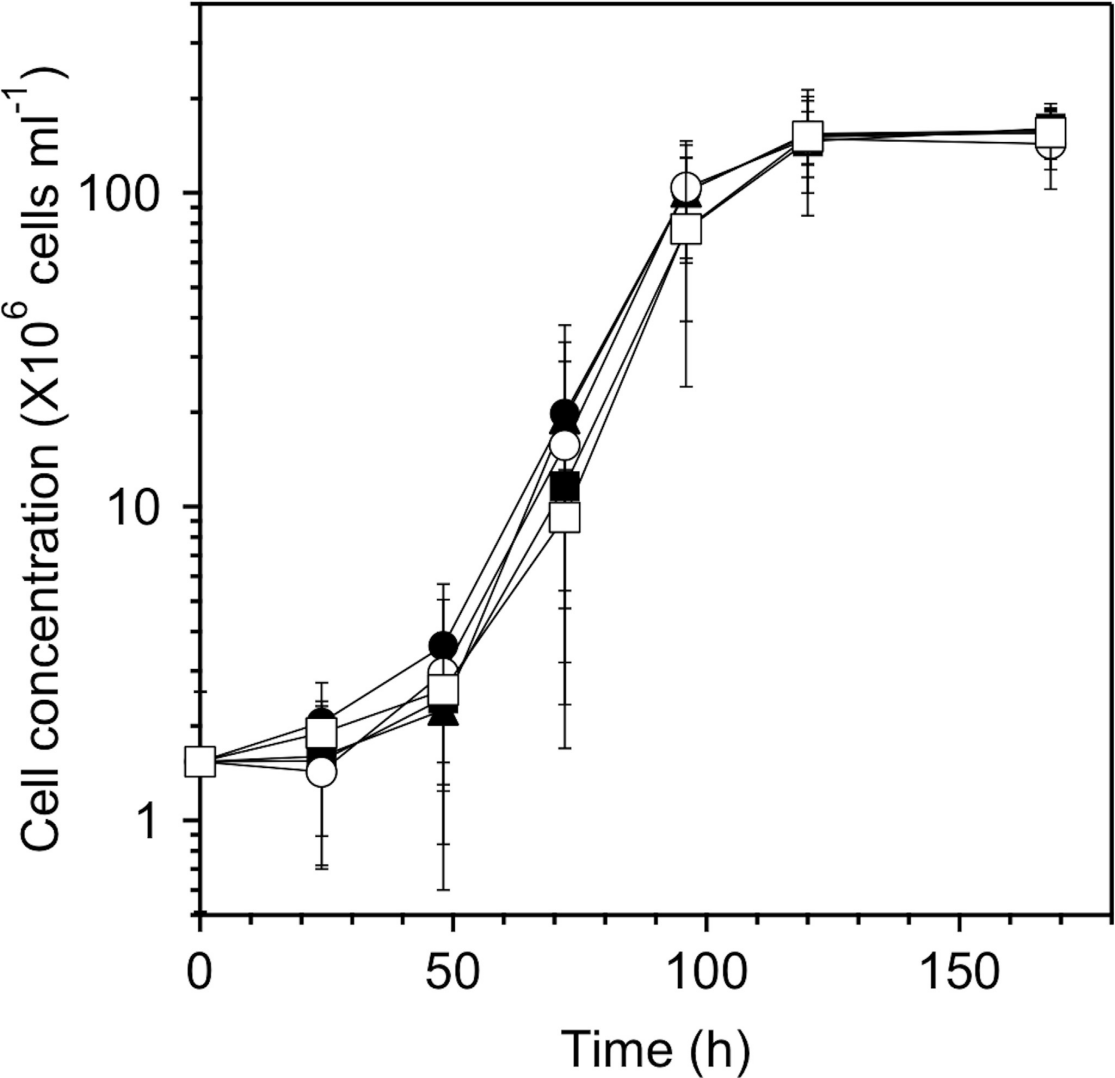
Effect of PEGs added to the culture medium on the growth of *M*. *magnetotacticum* MS-1. The cell concentration was evaluated, counting directly the number of cells in the culture medium using a Bacteria Counting Chamber. The closed circles, squares and triangles, and open circles and squares, respectively, correspond to the growth curves in the absence of PEG and in the presence of PEG200, PEG6,000, PEG20,000, and PEG500,000. The average values were calculated from three independent experiments. The error bars represent the standard deviations.

**Fig 2:**
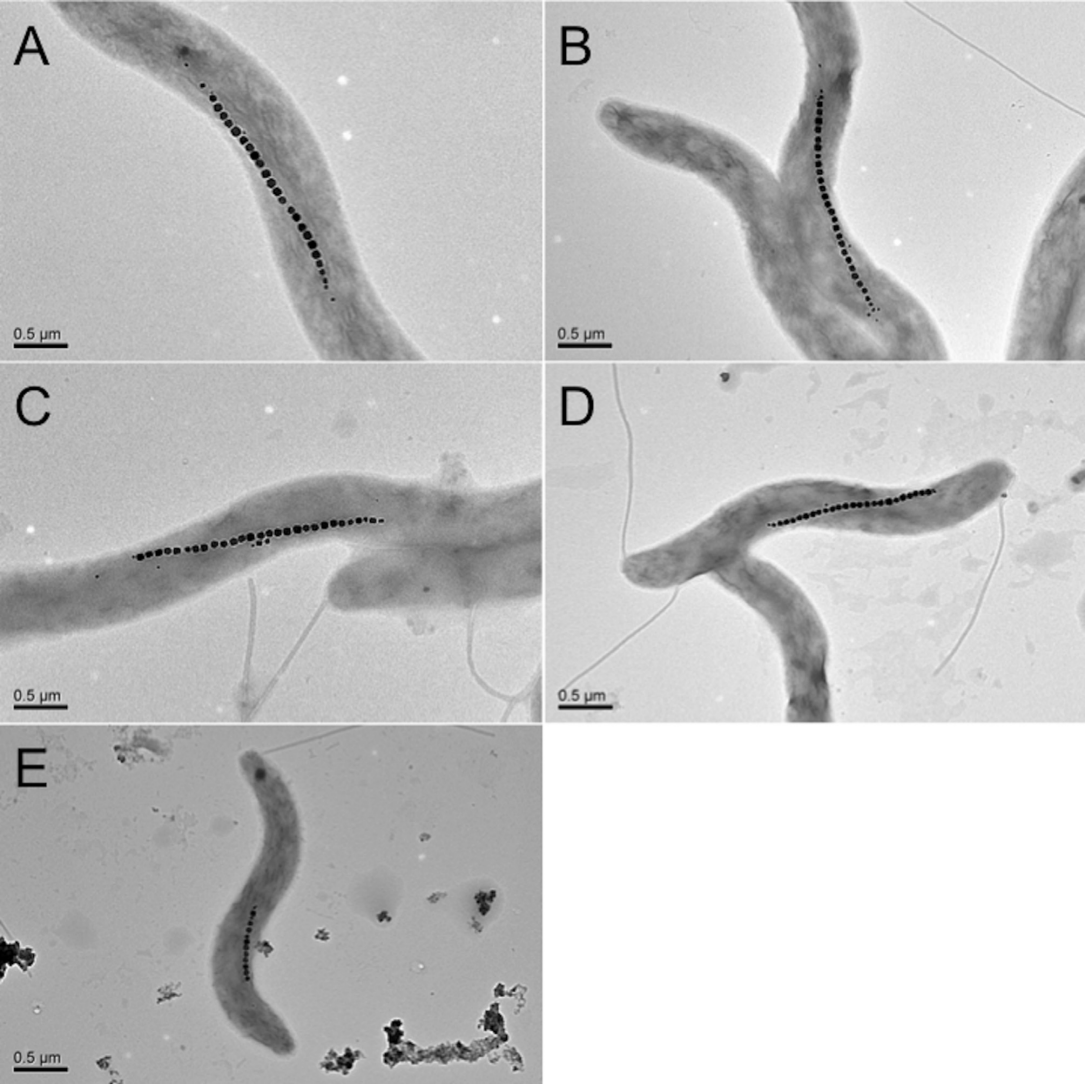
TEM images of magnetosomes synthesized in *M*. *magnetotacticum* MS-1 incubated in the culture medium containing PEGs. (A) Magnetosomes synthesized in the absence of PEG; (B), (C), (D), (E) Magnetosomes synthesized in the presence of 0.5% PEG200, 0.5% PEG6,000, 0.5% PEG20,000, and 0.5% PEG500,000. The scale bars represent 0.5 μm.

**Fig 3:**
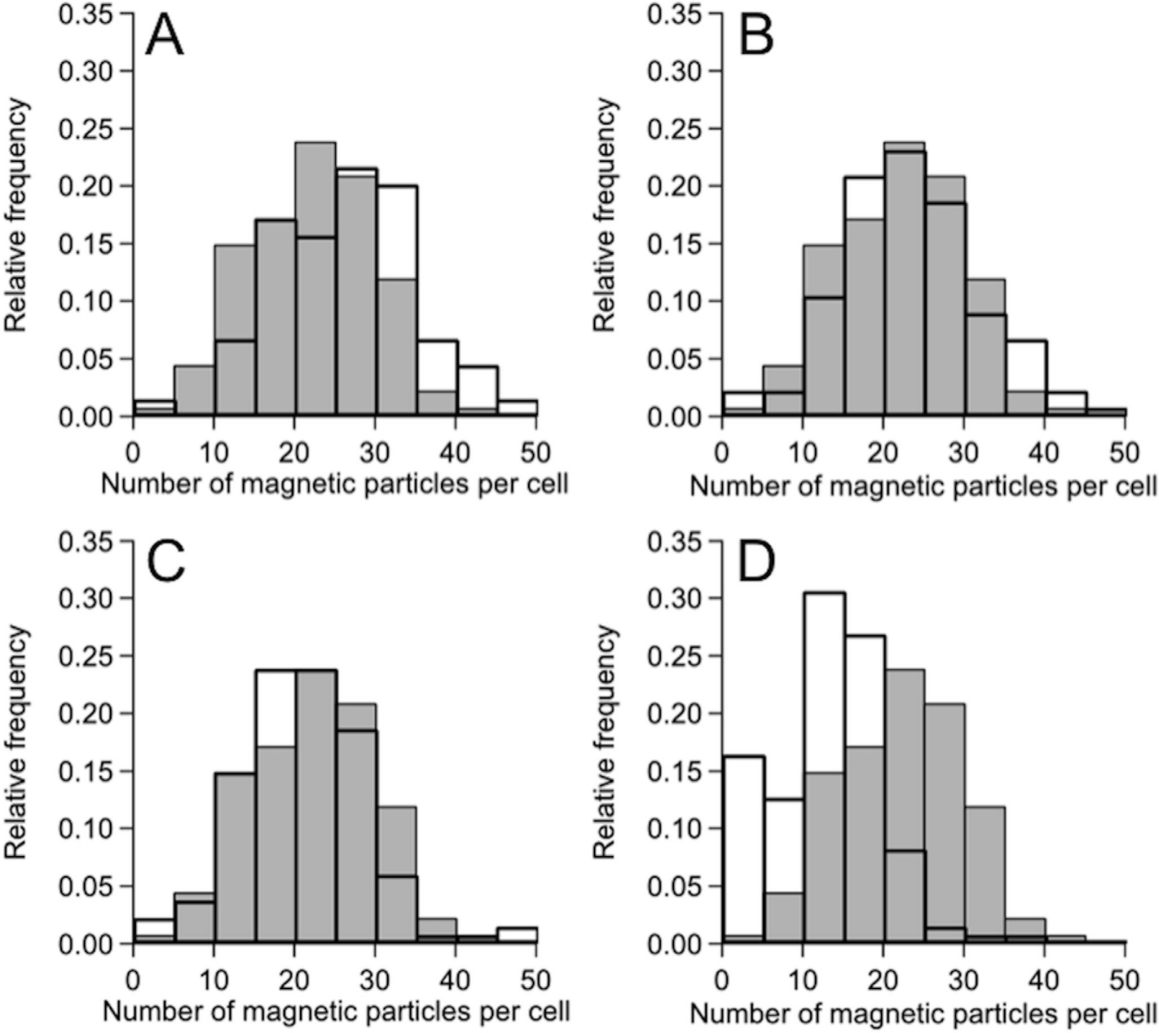
Distribution of the number of magnetosomes in each cell grown in the culture medium containing PEGs. (A), (B), (C), (D) Magnetosomes synthesized in the presence of 0.5% PEG200, 0.5% PEG6,000, 0.5% PEG20,000, and 0.5% PEG500,000. The histograms represent the number distributions of magnetosomes in the absence (gray bars) and presence (solid bars) of PEGs.

The growth curves of MS-1 cells in the culture medium in the absence of PEG and in the presence of 0.5% PEG200, 0.5% PEG6,000, 0.5% PEG20,000, and 0.5% PEG500,000 are shown in Fig 1. The final cell concentration reached (1.55 ± 0.27) × 10^8^, (1.60 ± 0.25) × 10^8^, (1.58 ± 0.28) × 10^8^, (1.43 ± 0.40) × 10^8^, and (1.56 ± 0.37) × 10^8^ cells ml^-1^, respectively (see Table 1). Thus, it was clearly shown that PEG200, PEG6,000, PEG20,000, and PEG500,000 hardly inhibited the cell growth and what is more, MS-1 cells formed magnetosomes under all of the above cultural conditions according to electron microscopy (see Fig 2). Note however that the size of the cells was smaller in the presence of PEG20,000 and PEG500,000 in the culture medium than that in the presence of PEG200 and PEG6,000, and that the addition of PEG500,000 discouraged the formation of magnetosomes. The number and size distribution of magnetosomes in each cell were determined from TEM images. The average number of magnetosomes per cell without PEG and with PEG200, PEG6,000, PEG20,000, and PEG500,000 were, respectively, 17.6, 21.8, 17.4, 14.2, and 7.2 (see Table 1 and Fig 3). There was approximately 24% increase in the average number of magnetosomes in the case of PEG200 (Fig 3A). Obviously, the culture medium containing PEG200 enhanced the synthesis of magnetosomes. Furthermore, the size of magnetosomes synthesized in the presence of PEG200 was larger than that in the absence of PEG200 by approximately 10%, noting that 34.1 ± 7.9 nm in the former case, while 30.9 ± 7.3 nm in the latter (see S2 Fig).

**Table 1:** Effect of PEGs on the growth of *M*. *magnetotacticum* MS-1 and the formation of magnetosomes.

Added substance	Time(h)	Final cell concentration* (×10^8^ cells ml^-1^)	Average number of magnetosomes per cell**	Normalized production rate of magnetosome***
None	168	1.55 ± 0.27	17.6	1.00
PEG200	168	1.60 ± 0.25	21.8	1.28
PEG6,000	168	1.58 ± 0.28	17.4	1.01
PEG20,000	168	1.43 ± 0.40	14.2	0.74
PEG500,000	168	1.56 ± 0.37	7.2	0.41

* The cell concentration was evaluated, counting directly the number of cells in the culture medium using a Bacteria Counting Chamber.

** The average values were obtained from two independent experiments.

*** Normalized production rate of magnetosomes *P* was defined by
$$P=C_{p}\times N_{p}{}_{}/\;(C_{0}\times N_{0}),$$
where *C*_*p*_ and *N*_*p*_ are, respectively, the final cell concentration and the average number of magnetosomes synthesized in each cell in the presence of PEG, whereas *C*_0_ and *N*_0_ are those in the absence of PEG.

The magnetic properties of MS-1 cells grown in the culture medium containing PEG200 were measured using a SQUID at 300 K. The mass magnetization is shown in S3 Fig. The saturation mass magnetization, residual mass magnetization and coercivity of strain MS-1 cultivated in the absence of PEG200 were 1.07 A m^2^ kg^-1^, 0.20 A m^2^ kg^-1^ and 11690 A m^-1^, whereas those in the presence of PEG200 were 1.15 Am^2^ kg^-11^, 0.23 A m^2^ kg^-1^ and 8680 A m^-1^. In other words, the magnetosomes showed soft ferromagnetic characteristics and the saturation mass magnetization increased by approximately 7% by adding PEG200 to the culture medium. It is supposed that the enhancement of the saturation mass magnetization in the presence of PEG200 might have been caused mainly by the increase in the average number of magnetosomes per cell (see Fig 3A), noting that the magnetization can also be changed by the alteration in the crystallinity and the size and number of magnetic domains in the particles, analyses of which were however beyond the scope of the present study. It is supposed that the magnetosomes synthesized in MS-1, which had been cultivated in the presence of PEG200, were well crystallized as in the case of those cultivated in the absence of PEG200, judging by the magnetic characteristics shown in S3 Fig and the previous reports on nanoparticles composed of magnetite (Fe_3_O_4_) [22–25].

In summary, the effect of PEG molecules added to the culture medium on the growth of MS-1 and the formation of magnetosomes was investigated for the first time and we found that the yield of magnetosomes was successfully increased by approximately 30% by adding 0.5% PEG200 to the culture medium. It is known that the lipid composition in the cell membranes of Gram-negative bacteria is altered by PEG [26] and that the activation of some membrane transport proteins in the bacterial membranes is affected by the changes in the lipid membrane composition [27]. In the MTB species, the activation of uptake of iron ions into the membrane vesicles is definitely required for the accumulation of iron ions in the magnetosome membrane vesicles and it was suggested that some membrane transport channel proteins may be playing an important role for the formation of magnetosomes in Magnetospirillum species [28–30]. Although the actual mechanism of the enhancement of the yield of magnetosomes induced by the addition of PEG200 to the culture medium has not yet been clearly understood, we suppose that PEG200 may promote the uptake of iron ions via the activation of some transport proteins in the magnetosome membranes, knowing that PEG200 permeates the cell walls thanks to its low molecular weight [26]. Since PEG200 is non-toxic and low-cost [31], the present methodology may well be utilized for the production of magnetosomes in large-scale bioreactors. We suppose that mass production of magnetosomes may eventually become possible by discovering appropriate molecules from the point of view of efficient transport of iron ions through the magnetosome membrane vesicles.

## Supporting Information

S1 Fig Number of magnetosomes in each cell grown in the culture medium supplemented with PEG6,000. (TIFF)

S2 Fig Distribution of the size of magnetosomes in each cell grown in the culture medium supplemented with PEG200. The histograms represent the size distributions of magnetosomes in the absence (gray bars) and presence (solid bars) of PEG200.
(TIFF)

S3 Fig Mass magnetization of magnetosomes synthesized in *M*. *magnetotacticum* MS-1 cultivated in the culture medium in the absence (black circles) and presence (white circles) of PEG200. (TIFF)

S1 Table Effect of several substances added to the culture medium on the growth of *M*. *magnetotacticum* MS-1 and the formation of magnetosomes. (DOCX)

S1 Text Effect of substances added to the culture medium on the growth of *M*. *magnetotacticum* MS-1 and the formation of magnetosomes. (DOCX)

S2 Text Number of magnetosomes in each cell grown in the culture medium. (DOCX)
