# Supplementary figures and images for: Effect of Polyethylene Glycol on the Formation of Magnetic Nanoparticles Synthesized by Magnetospirillum magnetotacticum MS-1
Source: PLoS One. 2015 May 20;10(5):e0127481. doi: 10.1371/journal.pone.0127481 (PMC4439050; doi:10.1371/journal.pone.0127481)

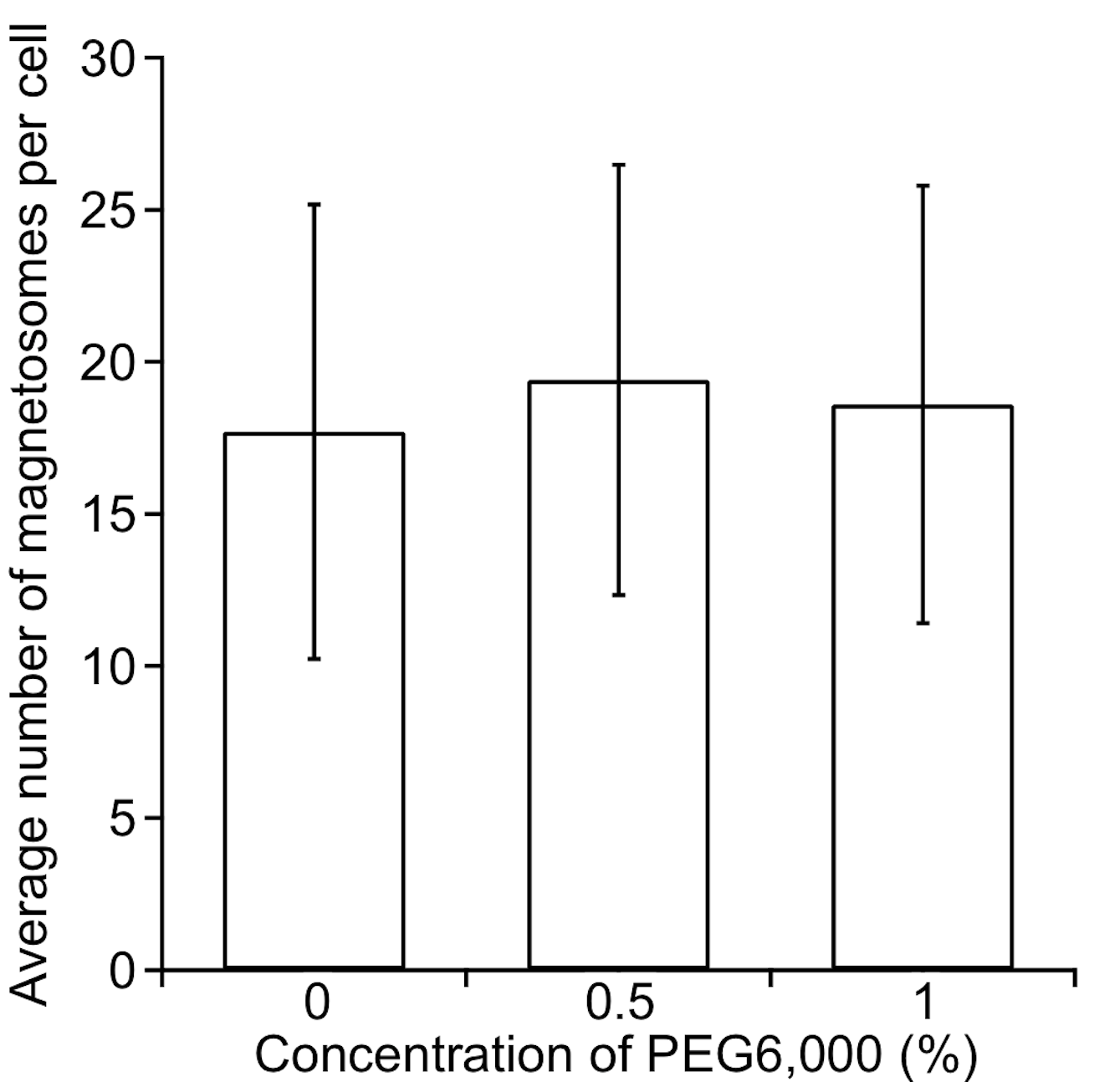

Supplement: S1 Fig — (TIFF) [file pone.0127481.s001.tiff]

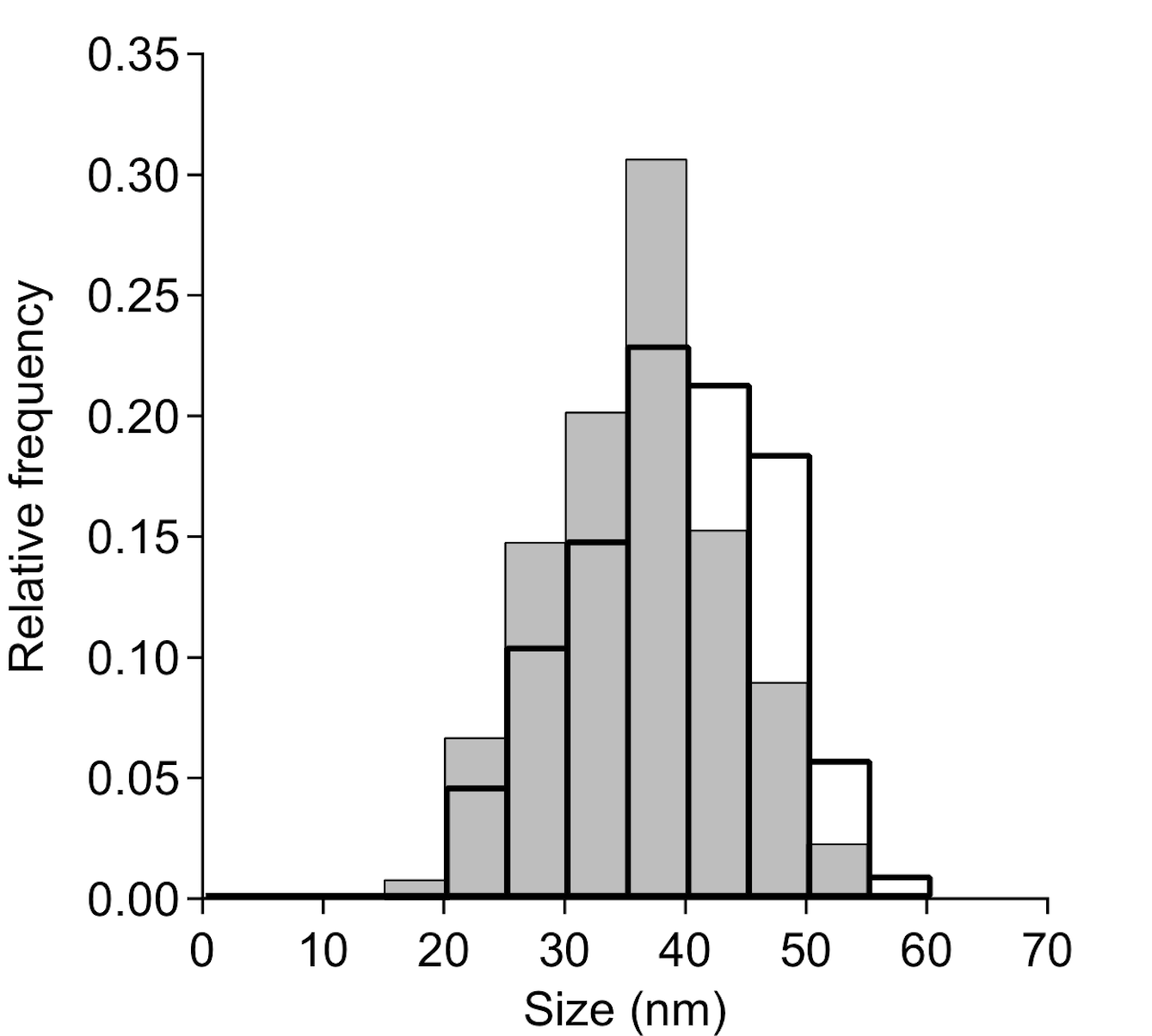

Supplement: S2 Fig — The histograms represent the size distributions of magnetosomes in the absence (gray bars) and presence (solid bars) of PEG200. (TIFF) [file pone.0127481.s002.tiff]

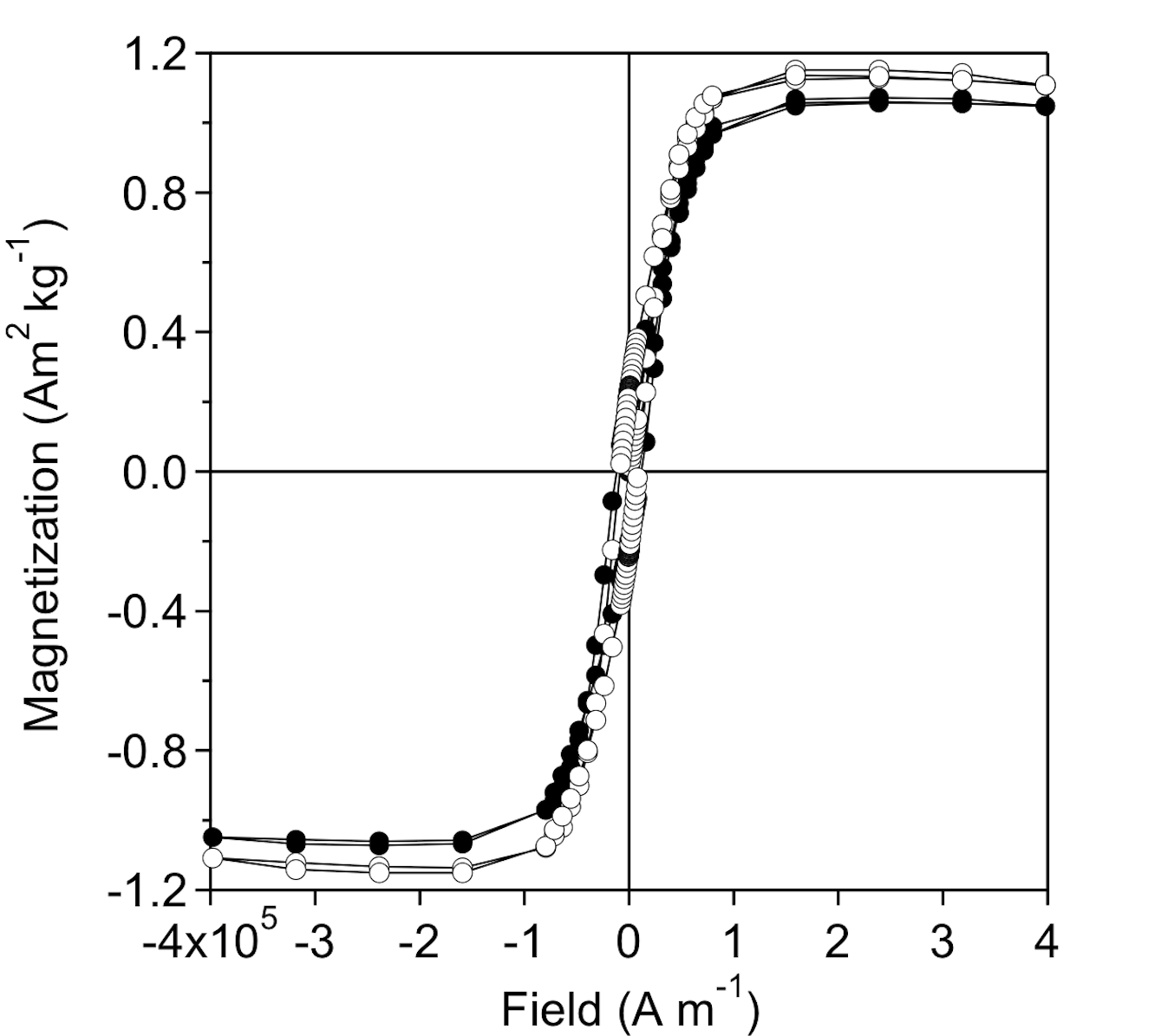

Supplement: S3 Fig — (TIFF) [file pone.0127481.s003.tiff]
