# Supplementary material for: Effect of Polyethylene Glycol on the Formation of Magnetic Nanoparticles Synthesized by Magnetospirillum magnetotacticum MS-1
Source: PLoS One. 2015 May 20;10(5):e0127481. doi: 10.1371/journal.pone.0127481 (PMC4439050; doi:10.1371/journal.pone.0127481)
Supplement: S1 Table — (DOCX) [file pone.0127481.s004.docx]

**S1 Table. Effect of several substances added to the culture medium on the growth of *M. magnetotacticum* MS-1 and the formation of magnetosomes.**

| **Added substances** | **Concentration (%)** | **Time (h)** | **Normalized final cell concentration^*^** | **Magnetic response** | **Normalized rate of the formation of magnetosomes**^**^ |
| --- | --- | --- | --- | --- | --- |
| *n*-Dodecane | 2.2 | 290 | 0.18 | + | - |
| *n*-Decane | 2.2 | 290 | 0.21 | + | - |
| *n*-Nonane | 2.2 | 290 | 0.03 | − | - |
| *n*-Octane | 2.2 | 290 | 0.03 | − | - |
| Cyclooctane | 2.2 | 290 | 0.03 | − | - |
| Diphenylether | 2.2 | 290 | 0.03 | − | - |
| *n*-Hexane | 2.2 | 290 | 0.03 | − | - |
| *n*-Octanol | 0.01 | 290 | 0.05 | − | - |
| PEG6,000 | 0.5 | 290 | 1.24 | + | 1.10 |
|  | 1.0 | 290 | 1.03 | + | 1.07 |
| Triton-X100 | 0.5 | 290 | 0.46 | + | - |
|  | 1.0 | 290 | 0.36 | + | - |
| Oleic acid | 0.5 | 290 | 0.30 | − | - |
|  | 1.0 | 290 | 0.15 | − | - |
| Olive oil | 0.5 | 290 | 0.79 | + | 1.11 |
|  | 1.0 | 290 | 0.88 | + | 1.13 |
| Glycerol | 0.5 | 290 | 1.02 | + | 1.01 |
|  | 1.0 | 290 | 0.85 | + | 1.00 |
| Soluble starch | 0.1 | 290 | 0.94 | + | 0.93 |
| CMC | 0.1 | 290 | 1.00 | + | 0.71 |
| Pectin | 0.1 | 290 | 0.40 | − | - |
| PEG6,000  + olive oil | 0.5  1.0 | 290 | 1.03 | + | 1.10 |

^*^ The cell density in the culture medium was evaluated, measuring the absorbance of photons of 600 nm wavelength.

^**^ The rate of the formation of magnetosomes was evaluated only when more than 10 magnetosomes were formed in a cell.
