# Supplementary material for: Effect of Polyethylene Glycol on the Formation of Magnetic Nanoparticles Synthesized by Magnetospirillum magnetotacticum MS-1
Source: PLoS One. 2015 May 20;10(5):e0127481. doi: 10.1371/journal.pone.0127481 (PMC4439050; doi:10.1371/journal.pone.0127481)
Supplement: S1 Text — (DOCX) [file pone.0127481.s005.docx]

**S1 Text. Effect of substances added to the culture medium on the growth of *M. magnetotacticum* MS-1 and the formation of magnetosomes.**

Strain MS-1 was grown in the presence of various additional substances (see S1 Table), assuming that they may affect the structures of the membranes of the cells and/or magnetosomes and as a result, the growth of MS-1 cells and the formation of magnetosomes may be altered. It is known that the cell envelopes are composed of polysaccharides, proteins, and phospholipids such as phosphate, fatty acids and glycerol, and that organic solvents and surfactants generally deteriorate the structure of the microbial membranes [1]-[3]. The culture medium containing *n*-nonane, *n*-octane, cyclooctane, diphenylether, *n*-hexane, and *n*-octanol completely inhibited the cell growth as expected. Although the addition of *n*-dodecane and *n*-decane to the culture medium strongly inhibited the growth of strain MS-1, the cells slightly responded to the external magnetic field. The inhibitory effect on the growth of the cells was obviously caused by the toxicity of organic solvents, which can be quantitatively evaluated by the LogP value [4]. We found that strain MS-1 is extremely weak against the organic solvents unlike most of the Gram-negative bacteria. The addition of PEG6,000 to the culture medium slightly increased the cell concentration and the rate of the formation of magnetosomes. The growth of strain MS-1 was suppressed in the culture medium containing Triton-X100 and oleic acid. The inhibitory effect was intensified with an increase in the concentration of Triton-X100 and oleic acid. Interestingly, strain MS-1 did not show any magnetic response in the culture medium containing oleic acid. Note that it is known that in green algae, an ionic current through the ionic channels in the plasma membranes is suppressed with the addition of oleic acid [5]. We therefore suppose that the ionic current was also prevented by the addition of oleic acid to strain MS-1, resulted in an insufficient supply of ferric ions to the membrane vesicles for the formation of magnetosomes. Olive oil slightly increased the rate of the formation of magnetosomes, but it decreased the cell concentration. When glycerol was added to the culture medium, no significant difference was obtained. Soluble starch slightly decreased the cell concentration and the rate of the formation of magnetosomes. CMC hardly inhibited the cell growth, but it decreased the rate of the formation of magnetosomes. Pectin inhibited the cell growth. These metabolizable substrates might have affected the nutritional and redox status of strain MS-1. We need to investigate the enzymes produced by strain MS-1 to understand the cause of the inhibition of the formation of magnetosomes in more detail. Since the addition of PEG6,000 and olive oil to the culture medium improved the cell growth and/or the rate of the formation of magnetosomes, we examined the synergistic effect of PEG6,000 and olive oil on the growth of strain MS-1 and the formation rate of magnetosomes by adding them together to the culture medium. However, no synergistic effect was observed on the growth of the cells and the rate of the formation of magnetosomes.

1. Aono R, Kobayashi H, Joblin KN, Horikoshi K. (1994) Effects of organic solvents on growth of *Escherichia coli* K-12. Biosci. Biotechnol. Biochem. 58: 2009–2014.
2. Favre-Bulle O, Schouten T, Kingma J, Witholt B. (1991) Bioconversion of n-octane to octanoic acid by a recombinant *Escherichia coli* cultured in a two-liquid phase bioreactor. Bio/Technology 9: 367–371.
3. Tilby MJ. (1978) Detergent-resistant variants of Bacillus subtilis with reduced cell diameter. J. Bacteriol. 136: 10-18.
4. Inoue A. Horikoshi K. (1989) A *Pseudomonas* thrives in high concentrations of toluene. Nature 227: 264–265.
5. Kataev AA, Zherelova OM, Grishchenko VM. (2013) Effects of oleic acid on ionic channels of plasma membranes of green alga *Chara corallina*. Biochemistry (Moscow) Supplement Series A: Membrane and Cell Biology.7: 141-147.
