# Supplementary material for: Effect of Polyethylene Glycol on the Formation of Magnetic Nanoparticles Synthesized by Magnetospirillum magnetotacticum MS-1
Source: PLoS One. 2015 May 20;10(5):e0127481. doi: 10.1371/journal.pone.0127481 (PMC4439050; doi:10.1371/journal.pone.0127481)
Supplement: S2 Text — (DOCX) [file pone.0127481.s006.docx]

**S2 Text. Number of magnetosomes in each cell grown in the culture medium.**

The effect of PEG6,000 added to the culture medium on the synthesis of magnetosomes was investigated, counting the number of magnetosomes synthesized in each cell, which had been cultivated in the presence of 0.5 and 1.0 % PEG6,000. S1 Fig. shows the average number of magnetosomes synthesized in each cell. The average number of magnetosomes was, respectively, 17.7, 19.4, and 18.6 per cell in the case of 0, 0.5, and 1.0 % PEG6,000.
